# A morphometric and histological study of placental malaria shows significant changes to villous architecture in both *Plasmodium falciparum* and *Plasmodium vivax* infection

**DOI:** 10.1186/1475-2875-13-4

**Published:** 2014-01-04

**Authors:** Sethawud Chaikitgosiyakul, Marcus J Rijken, Atis Muehlenbachs, Sue J Lee, Urai Chaisri, Parnpen Viriyavejakul, Gareth D Turner, Emsri Pongponratn, Francois Nosten, Rose McGready

**Affiliations:** 1 Department of Tropical Pathology, Faculty of Tropical Medicine, Mahidol University, 420/6 Rajvithi Road, Bangkok 10400, Thailand; 2 Mahidol-Oxford Research Unit, Faculty of Tropical Medicine, Mahidol University, 420/6 Rajvithi Road, Bangkok 10400, Thailand; 3 Shoklo Malaria Research Unit, Mae Sot, Thailand; 4 Department of Pathology, University of Washington, Seattle, WA 91895, USA; 5 Centre for Tropical Medicine, Nuffield Department of Clinical Medicine, University of Oxford, Oxford OX3 7LJ, UK

**Keywords:** Malaria, Placenta, Pregnancy, Pathophysiology, Morphometry, Image analysis, Villi

## Abstract

**Background:**

Malaria in pregnancy remains a major health problem. Placental malaria infection may cause pathophysiological changes in pregnancy and result in morphological changes to placental villi. Quantitative histomorphological image analysis of placental biopsies was performed to compare placental villous architecture between active or treated placental malaria cases and controls.

**Methods:**

A total of 67 placentas were studied from three clinical groups: control patients who did not have malaria (n = 27), active (n = 14) and treated (n=26) malaria cases, including both *Plasmodium falciparum* and *Plasmodium vivax* infections. Image analysis of histological placental sections was performed using ImageJ software to measure the number and size (area) of terminal villi, perimeter measurement per villus and total perimeter per unit area, and number of capillaries per villus (vascularity). Histological features of placental malaria were scored and these results were correlated with malaria status and clinical outcomes.

**Results:**

Villous size correlated with vascularity (p <0.0001) but was inversely correlated with observed villi per unit area, (p = 0.0001). Significantly greater villous area and vascularity was observed in UK controls. Indices of histological malaria infection were significantly greater in active versus treated malaria cases. Active placental malaria cases showed significantly smaller villous area (p <0.0084), vascularity (p <0.0139) and perimeter (p <0.0006) than treated malaria cases or controls, but significantly more villi per unit area (p <0.0001). Villous size in treated malaria cases was significantly larger than active placental malaria cases (p <0.001) and similar to controls. There was a significant relationship between villous number and anaemia at the time of infection (p <0.0034), but not placental weight, birth weight or gestational age at delivery. No differences were found between histology or villous morphology comparing infections with *P. falciparum* or *P. vivax*.

**Conclusions:**

These results imply that villous size, perimeter and vascularity are acutely decreased during active placental malaria, decreasing the surface area available for gas exchange per villus. However the increased number of villi per unit area offsets this change and persists after treatment. Histopathological and villous architectural changes may be reversed by early detection and appropriate anti-malarial treatment.

## Background

Malaria infection during pregnancy is a major public health problem in tropical and subtropical regions throughout the world, with substantial risks for the mother, her foetus and subsequently the neonate [1]. Malaria in pregnancy, defined as malaria infection in the placenta or maternal blood at any time during gestation, is associated with a number of materno-foetal complications, such as intra-uterine growth restriction (IUGR), anaemia and pregnancy loss [2-5]. The structure of the placental villous tree changes during normal placental development due to major physiological changes occurring in the placenta during maturation, reflected in changes to the morphology of the stem, intermediate or terminal villi and blood vessels within them [6-8]. Placental malaria, namely the presence of parasitized erythrocytes in the placenta, is inferred to occur during maternal malaria infection, but is only histologically confirmed if parasites are present on histological examination of the placenta following delivery.

Placental malaria infection causes pathological changes such as inflammation, intervillous fibrin deposition and infarction, that can persist until delivery and affect the function of the materno-placental unit, contributing to adverse birth outcomes and increased perinatal mortality and morbidity [9]. However the effect of malaria infection on villous architecture has not been studied in depth. Previous studies have used histomorphometric image analysis to examine placental villous architecture in other diseases causing IUGR, including pre-eclampsia (PE) and diabetes [10-16]. There are a number of physiological factors that can affect villous architecture, as measured on image analysis. Both the age and parity of the mother have been shown to affect villous numbers and vascularity. For instance, the volume density of capillaries in terminal villi was shown to be significantly lower in older women in comparison to the placenta in younger mothers [7]. In idiopathic IUGR, the number of capillaries in the terminal villi and the villous area was significantly decreased compared with controls appropriate for gestational age group [10,11]. In PE, the villous area and vascularity were significantly smaller than controls [13]. This study aimed to examine villous architecture and vascularity in placental malaria, relate these to clinical outcomes and the patterns of histopathological changes that have been characterized previously in malaria. It also sought to determine whether any such changes differed between infections with either *Plasmodium falciparum* and *Plasmodium vivax*, or were ameliorated by effective anti-malarial treatment before delivery.

## Methods

### Ethical approvals

Ethical permission for collection of placental biopsies in the specific studies was granted with each separate study [17-21]. Use of these data and specimens in this morphometric study was approved by the Ethics Committee of Faculty of Tropical Medicine, Mahidol University (TMEC 12–027).

### Patient selection and clinical groups

In this study a total of 67 placental biopsies were examined, from cases that were divided into three main clinical groups – non-malaria controls (n = 27), active placental malaria cases (n = 14) and treated malaria cases (n = 26).

**Figure 1:**
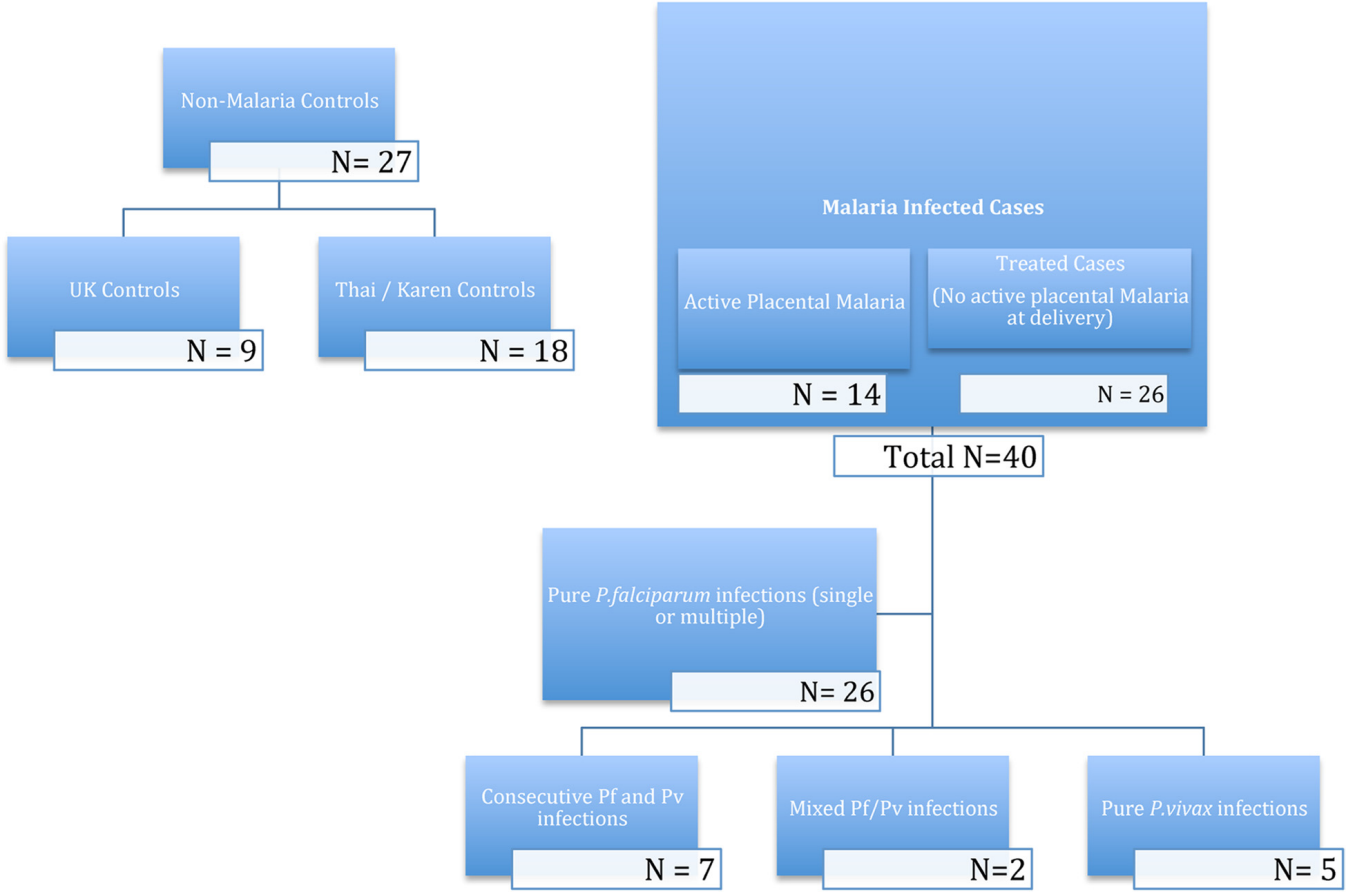
Flow chart showing the type and number of the cases in active and treated malaria and control groups.

The active or treated malaria-infected patients (n = 40) included n = 26 who had only *P. falciparum* infection, n = 5 with mono-infection by *P. vivax* and n = 9 with consecutive or mixed *P. falciparu*m/*P.vivax* infections. Of this latter group, n = 7 had multiple consecutive infections, including at least one episode each with either *P. falciparum* and *P. vivax,* and two of whom had proven mixed infections with both organisms simultaneously. Details of the malaria status of the cases are shown in Figure 1.

Placental biopsies were collected between 2004–2011, from the Department of Tropical Pathology, Faculty of Tropical Medicine and three previously published studies investigating malaria in pregnancy conducted at Shoklo Malaria Research Unit (SMRU) field research station at Mae Sot, Thailand. These were studies of the histopathology of placental malaria, used to identify cases with acute infection at delivery with both *P. falciparum* and *P. vivax*[17], an observational ultrasound in pregnancy study (UPS) [18,19] and the artemether-lumefantrine treatment of malaria study (PCA) [20,21]. Women in these studies included ethnic Karen refugees and migrants from an area of approximately 120 km around the border between Thailand and Myanmar, surrounding the town of Mae Sot and the Maela refugee camp. They attended weekly antenatal clinics for malaria screening and all parasitaemic episodes were treated according to WHO guidelines. As a comparator group for villous morphology in a geographically distinct group of normal control non-malaria cases, a series of normal placental biopsies from the UK were also examined.

The cases included:

(i) Control normal placentas from UK women delivering at The John Radcliffe Hospital, Oxford, UK (n = 9), with no fever complicating pregnancy or delivery.

(ii) Control placentas from Karen women delivering at SMRU, who had no evidence of malaria infection by active screening by microscopy throughout pregnancy and negative histology at delivery (n = 18, from UPS study).

(iii) Active placental malaria cases (n = 14), where malaria was diagnosed by microscopy and subsequent blood films in the mother, and placenta biopsies confirmed acute placental malaria infection at the time of delivery (from histopathology and UPS studies).

(iv) Cases with known single or multiple malaria infections treated during pregnancy at various time points prior to delivery (n = 12, from UPS study, n = 3 of whom had active placental malaria at delivery and are therefore already included in group iii above).

(v) Single-treated malaria infection in early, middle or late pregnancy (from PCA) but not at delivery (n = 17), selected randomly from patients where mono-infection was proven and subsequent screening microscopy until delivery was negative [19]. ‘Early’ infection (n = 6) included women who had a clinical episode of malaria treated at least five months prior to delivery; ‘middle’ (n = 6) included those with malaria infection two to four months before delivery and ‘late’ infection (n = 5) those with treated malaria infection within one month before delivery.

Clinical outcome data on the patients included the gestational age at delivery (EGA), the placental weight at delivery (in grams), the birth weight of the baby (in grams), and the presence of maternal anaemia at the time of infection. This was measured using standard haematocrit (HCT) measurement, and stratified according to the criteria used at SMRU for defining maternal anaemia 1: HCT > 30% - normal, 2: HCT 25-30% - mild anaemia and HCT < 25% - severe anaemia. EGA was measured for all Mae Sot patients by dating ultrasound prior to 24 weeks, Dubowitz estimation of gestational age and the last menstrual period. Because malaria in this study group was diagnosed actively and prospectively, and treated when found regardless of symptoms, the possibility of asymptomatic placental infection at delivery was minimal. Each case was defined at delivery as being malaria negative on the basis of smear microscopy on cord blood, maternal blood and placental smears. Summary details of the clinical groups are shown in Table 1.

**Table 1:** Descriptive data summarising the different clinical groups by malaria status

**Clinical group**	**Diagnosis**	**Maternal age at delivery (years, mean +/− SD)**	**Estimated gestational age at delivery (weeks, mean +/− SD)**	**Median parity (range)**	**Median number of malaria episodes (range)**	**No. of cases with pure Pf N = 26 (% cases)**	**No. of cases with pure Pv N = 5 (% cases)**	**Mixed Pf + Pv N = 9 (% cases)**	**Mean placental weight at delivery/g (SD)**	**Birth weight/g (SD)**	**Haematocrit (%)**
**CONTROLS N = 27**	UK + Thai/Karen controls	29.6 (6.2)	38.2 (2.42)	2 (0–5)	0	-	-	-	493 (127)	2951 (457)^b^	30.1 (3.21)^b^
**TREATED MALARIA N = 26**	Malaria infection during pregnancy but not at delivery	28.0 (6.7)	39.6 (1.21)	2 (0–8)	1 (1–6)	17 (65.4%)	3 (11.5%)	6 (23.1%)	524 (130)	2923 (322)	27.0 (2.64)
**ACTIVE MALARIA N = 14**	Active placental malaria at delivery	24.7 (6.5)	39.1 (1.59)	0 (0–4)	1 (1–7)	9 (64.3%)	2 (14.3%)	3 (21.4%)	474 (116)	2801 (511)	27.2 (3.13)
**p value across groups**	-	0.084	0.09	0.048	0.83^a^	1.0^a^	1.0^a^	1.0^a^	0.56	0.692	0.0034*

^a^Comparison does not include controls; ^b^No data from UK controls, n = 9 * significant.

### Specimen preparation

Placental biopsy samples were taken by trained personnel according to established protocols, taking a full thickness biopsy including maternal and fetal surfaces, half way between the placental edge and the cord insertion site. Specimens were fixed in 10% neutral buffered formalin (UPS, and histopathology study and UK control patients) or Streck’s fixative (PCA study), then subsequently embedded in wax and processed using standard histological techniques to produce paraffin blocks. All processing and histological staining was performed in batches at the same pathology laboratory. Four μm histological sections were cut on a rotary microtome, and stained with both haematoxylin and eosin and Giemsa for histological examination.

### Image analysis of placental villi

The morphological study examined terminal, as opposed to stem or intermediate villi, because previous studies on the effects of disease on villous morphometry, and a pilot study in these cases, indicated that the predominant changes caused by disease are seen in these villi. Terminal villi were identified using a series of criteria including thinned trophoblastic lining, lack of muscularized arterioles, and more than 50% of the cross sectional area being occupied by vessels [8].

A total of 67 placenta specimens were used for image analysis by assessing four main parameters: the cross sectional surface area (size) of terminal villi; the number of capillaries per terminal villus; the number of terminal villi per high powered field (hpf); and the perimeter measurement of terminal villi, both the mean perimeter of individual villi and the total perimeter measurement in ten hpf.

Tissue sections were examined and digital images were captured from ten randomly selected ×400 hpf per slide using an Olympus DP-20 colour video camera interfaced with an Olympus BX41 microscope. The images were scanned at a resolution of 1,600 × 1,200 pixels using Olympus DP-20 BSW software (Version 2.2) and stored. Only the intermediate zone of the placenta was used and areas where villi could not be distinguished, with placental infarction, intervillous fibrin deposition, or arterial vessels forming the primary stem and intermediate villi were avoided, as these can distort the observed villous size [7,13]. Only terminal villi showing outlines completely within the microscopic field were counted. Captured images were used as raw data to quantify the villous architecture using the image analysis software ImageJ (Version 1.46, NIH, Maryland, USA) and to measure the area of terminal villi (μm^2^), number of capillaries per terminal villous and perimeter of terminal villi (μm).

### Area of the terminal villi (μm^2^)

**Figure 2:**
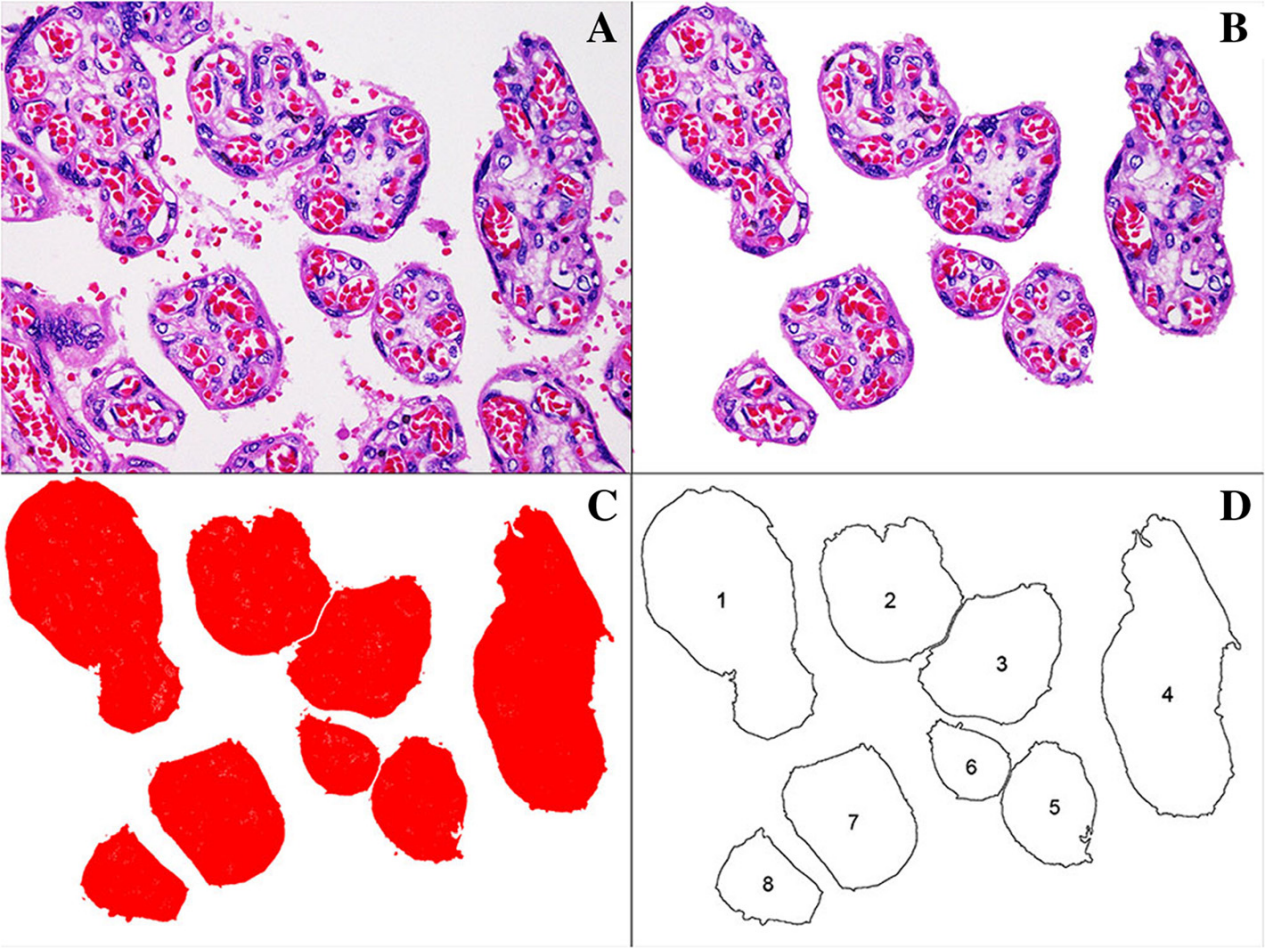
**Image processing steps used to generate quantitative outputs from haematoxyling and eosin-stained placental sections.** The various stages of processing are graphically illustrated in the following sequence: **(A)** original image (magnification x400); **(B)** preprocessing step following removal of partial outlines and non-terminal villi; **(C)** adjusted threshold to generate solid red pixelated areas; **(D)** computerized measurement of area of terminal villi using the resultant outlines.

The images were first subjected to a preprocessing step (Figure 2) which involved the removal of non-relevant areas to leave only single terminal villi outlines, using an eraser tool in Photoshop (Version CS6, Adobe System Inc., California, USA) software. Images were then adjusted by ImageJ to convert them to greyscale 8-bit images. After adjusting the threshold, the area of terminal villi was calculated as μm^2^ from the area of red pixels. The mean area of terminal villi for a case was calculated by the ratio between the total area and number of terminal villi in the field across all ten hpf.

### Number of capillaries per villous (villous vascularity)

The villous vascularity was measured using the cell counter tool in ImageJ to count the number of open capillary spaces, lined by endothelium, in each villous (regardless of whether this contained erythrocytes or not). The number of capillaries per villous was calculated by the total number of villous vessels divided by the number of terminal villi across ten separate hpf.

### Number of terminal villi per high-powered field (hpf)

The number of terminal villi in ten hpf was counted by eye with light microscopy in each case. The mean number of terminal villi per hpf in each patient was calculated by the ratio between the total number in ten hpf/ten. Results for individual cases were then combined for comparison between clinical groups.

### Perimeter measurement of villi

The perimeter of terminal villi (in μm) was measured in two ways. The mean perimeter measurement of individual villous cross sections (for all villi in ten hpf) was calculated automatically in a similar manner to the villous area by ImageJ, directly from the outlined edge of terminal villi (Figure 2D). The total perimeter measurement of all villous cross sections in ten hpf was also calculated.

### Histological scoring of changes in placental malaria infection

The scoring system of Muehlenbachs *et al*. [22] was used to grade the presence or absence of parasitized red blood cells (PRBC), the density of inflammation and the presence of haemozoin pigment in intervillous fibrin. Semiquantitative scores for inflammation and pigment were also generated for each case (the histoscore). When present, infected erythrocytes were used to estimate a parasitaemia of infection in the placenta by counting infected and uninfected red blood cells over 100 hpf and calculating the percentage of RBCs infected.

### Statistical analysis

The specimens were prepared and analysed blind to the clinical group. Analyses were performed using the statistical program SPSS version 18 (SPSS Inc, Chicago, Illinois, USA) for Windows and STATA v12.0 (StataCorp LP, College Station, TX, USA). Data were described using means (standard deviation-SD) and frequencies (%). Due to the relatively small sample size and unequal variances across groups, non-parametric tests were used for comparisons. Correlations between villous number, size and vascularity and between clinical groups, across all individuals and between clinical outcome measures, were assessed using Kendall’s rank correlation coefficient (tau). The relationship between histoscore parameters and measures of villous architecture between clinical groups, subgroups of the PCA cases, and with clinical outcomes such as anaemia, was performed using the Fisher’s exact test and the Kruskall-Wallis test. Differences were considered statistically significant for p-values less than 0.05. However, as this was an exploratory study, numerous comparisons were made and exact p-values (to the third decimal place) are reported so that the reader may apply Bonferroni corrections, if preferred (where the statistically significant p-value is calculated as α/n, where α = 0.05 and n = number of tests).

## Results

Maternal malaria in southeast Asia differs from the African setting in that the disease is not endemic, there is a substantial proportion of *P. vivax* as well as *P. falciparum* infections, and also mixed infection with both organisms. In addition the patients included in this study were frequently tested for malaria and treated when this was detected, so active placental malaria at delivery is rare and the duration of undiagnosed malaria infections during gestation is minimal [17]. In malaria-infected cases, including both active and treated patients (a total of eighty-six infective episodes) the mean maternal parasitaemia on diagnosis was 21,308/μl (+/− SD 102,150).

### Correlation between villous area, vascularity and villous number

**Figure 3:**
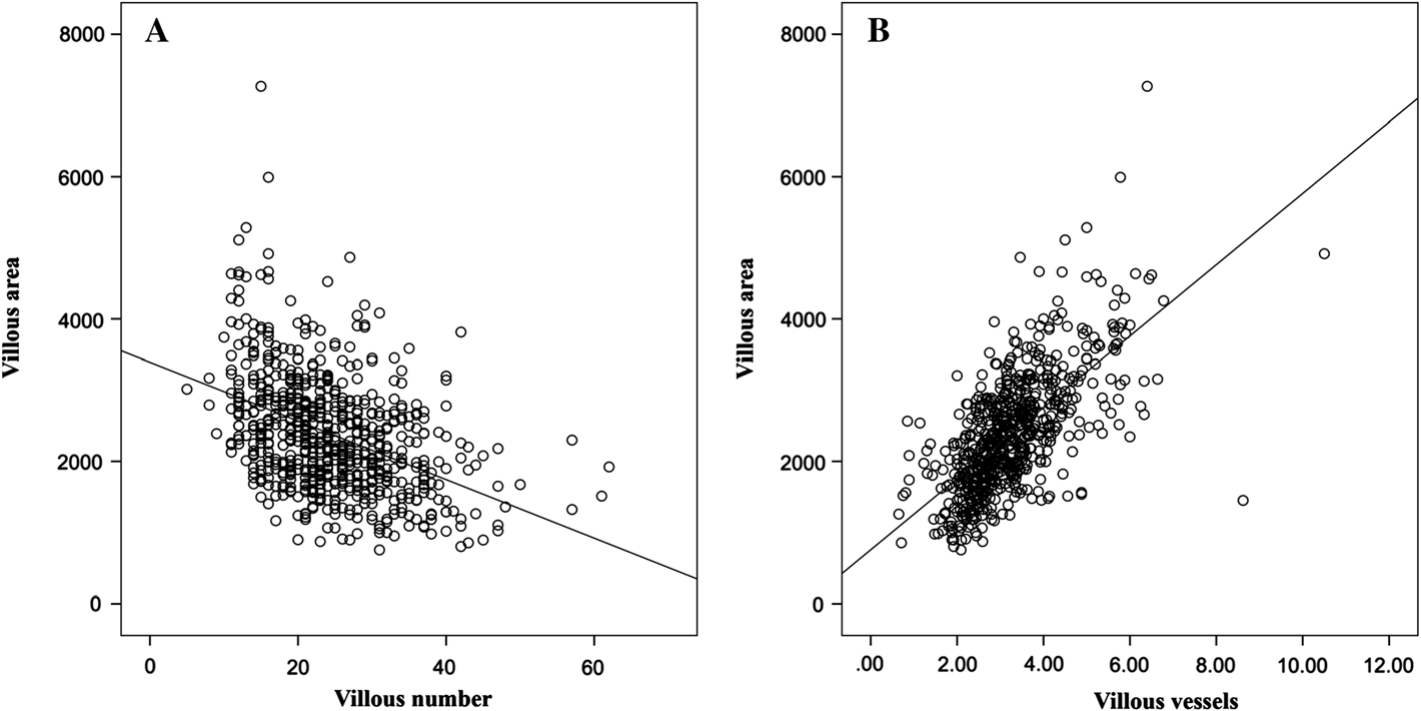
**Correlations between the villous area, number of villi per high-powered field and villous vascularity.** 3 **A** – Dot plot showing the negative correlation between the villous area and observed number of villi per hpf in individual cases (Line = line of best fit, Spearman rank correlation coefficient rho = −0.425, *p* <0.0001). 3 **B** – Dot plot showing the positive correlation between the villous area and villous vascularity in individual cases (Line = line of best fit, Spearman rho = 0.668, *p* <0.0001).

There was a significant inverse correlation between villous area and villous numbers observed per hpf (Kendall’s tau = −0.3216; p = 0.0001) either examining all cases or individual clinical groups (Figure 3A), because larger villi meant that less villi were seen per hpf at a given magnification. These two measurements were therefore covariate and not independent as an outcome measure of placental pathology. Conversely, there was a significant positive correlation between villous area and villous vascularity (Kendall’s tau = 0.4799; p <0.0001) (Figure 3B).

### Fixation and geographical origin influence observed villous architecture

**Figure 4:**
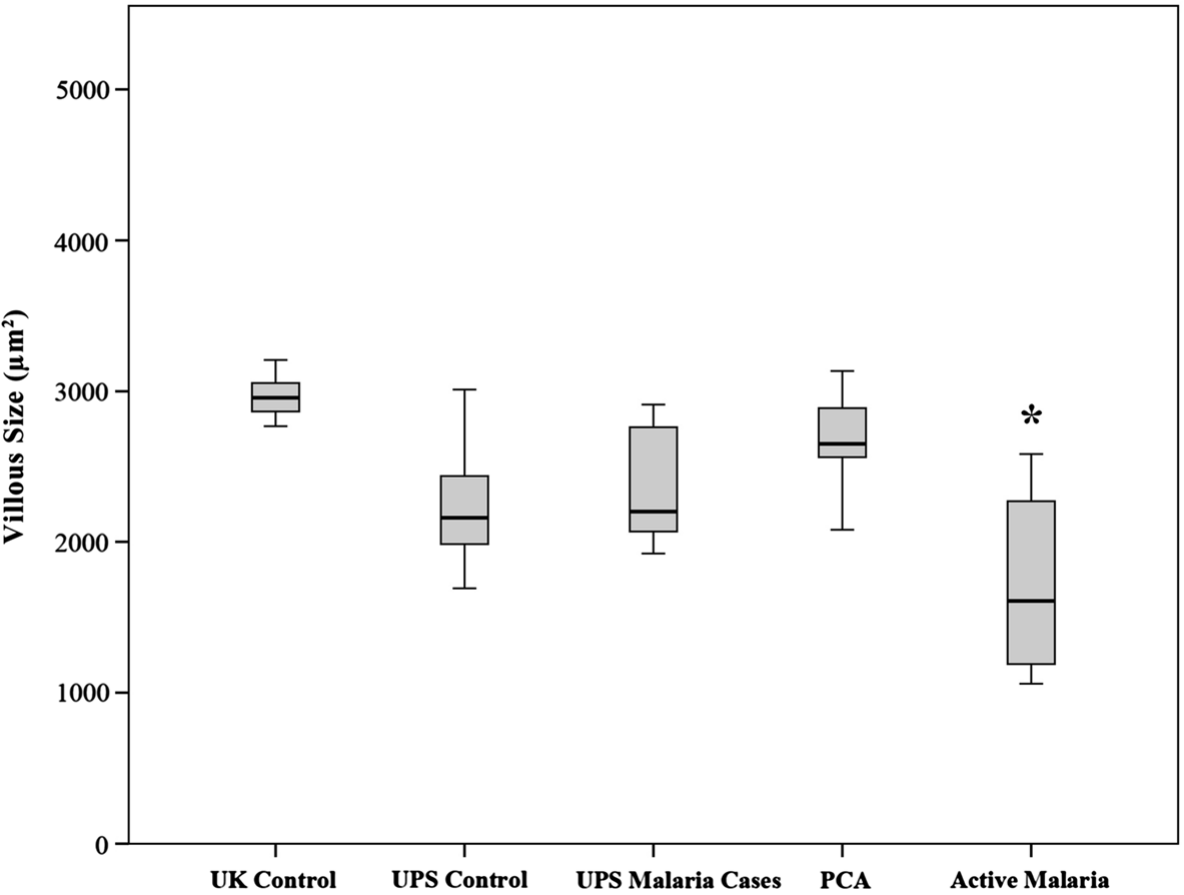
**Boxplot graph comparing villous area between clinical groups of controls and cases derived from different fixative methods or geographical origin.** A boxplot showing the distribution of villous area in sq μm between groups including UK controls, UPS cases (Thai/Karen cases fixed with formalin, divided into control non-malaria infected and malaria -infected UPS cases), PCA cases (Thai/Karen cases fixed with Streck fixative), and active placental malaria cases (Thai/Karen cases also fixed with formalin). UK controls showed significantly higher villous area than other groups (p <0.0004). PCA cases showed a non-significantly higher villous area than UPS controls fixed in formalin (p <0.0842). Active placental malaria cases showed significantly lower villous areas than all other groups *. Box and whisker plots for this and subsequent figures include; line = median, upper and lower bar = 25 and 75^th^ centiles, respectively, with shaded box covering interquartile range; whiskers cover maximum and minimum values.

Several other potentially confounding factors influenced the observed villous area. The first was the method of fixation. Non malaria-infected Thai/Karen cases fixed in formalin had a non-significantly lower mean villous area than cases fixed with Streck’s solution (p = 0.0842) but significantly lower villous vascularity (p <0.0002). In addition, the UK control group had a significantly greater villous area (p <0.0004) and villous vascularity (p <0.0087) than Thai/Karen control cases indicating a difference in observed villous area on a geographical basis (Figure 4). Other placental villous architectural features, such as number of villi/hpf and perimeter measurements, were not significantly different between the two control groups. One potential explanation for this might be villous congestion in UK deliveries due to delayed clamping of the umbilical cord, which was not practised in Karen women at the time these samples were collected. UK control cases had higher body weights than Karen controls, but the placental weights at delivery between these populations was not significantly different.

A post-hoc analysis of the results comparing UK controls, UPS, PCA and active malaria groups separately confirmed the significant differences in villous morphology across these groups, regardless of racial origin or fixation method. Therefore, despite the differences in villous architecture caused by the fixation method and geographical origin of the patient, outlined above, the analysis of the clinical groups compared combined control cases (UK and Thai/Karen controls; patients with no malaria infection) with all treated malaria cases (proven malaria infection at some point in gestation detected by screening and treated, but no observable PRBCs in the placenta at delivery) and active placental malaria cases (where histological evidence of parasites within the placenta confirmed acute malarial infection at delivery).

### Changes in villous architecture in malaria cases

**Figure 5:**
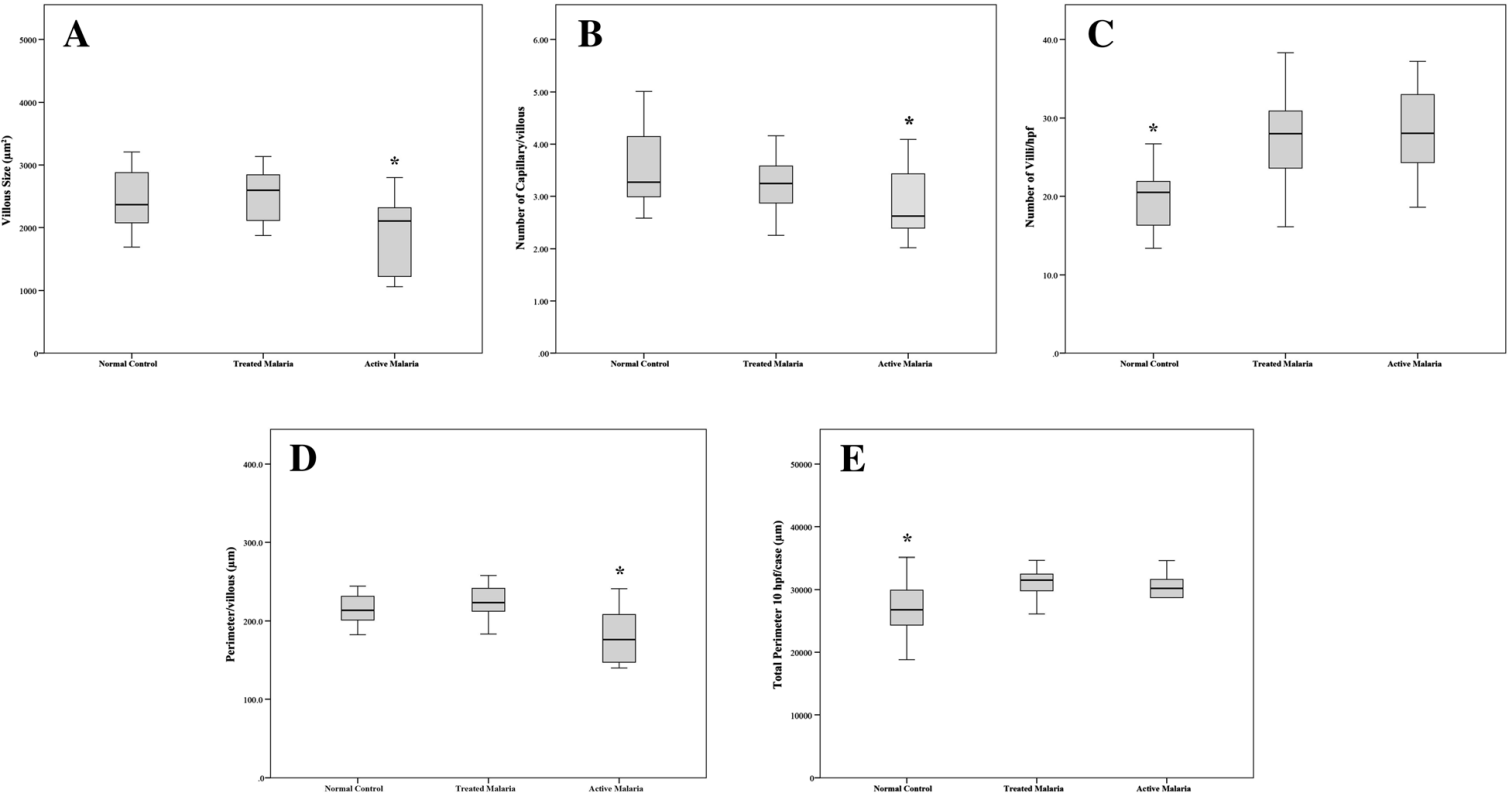
**Boxplot graphs showing variation in placental villous morphological parameters between clinical groups.** 5 **A** - Villous size. There was a significantly lower villus cross sectional area in active malaria cases compared to control or treated malaria (*p <0.0084, Fisher’s exact test). 5 **B** - Villous vascularity. There was a significantly lower number of capillaries per villus in active malaria cases compared to control or treated malaria (*p <0.0139, Fisher’s exact test). 5 **C**: Number of villi per high-powered field. There was a significantly higher number of villi seen per hpf in both active and treated malaria cases compared to controls (*p <0.0001, Fisher’s exact test). 5 **D**: Mean perimeter measurement per villus. There was a significantly lower perimeter measurement per individual villus seen in active malaria cases compared to treated malaria cases and controls (*p <0.0006, Fisher’s exact test). 5 **E** - Total perimeter measurement for all villi in 10 hpf. There was a significantly higher total perimeter measurement of all villi seen per 10 hpf in both active malaria and treated malaria cases compared to controls (*p <0.0004, Fisher’s exact test).

There was a significant decrease in villous area in active malaria-infected cases compared to both controls and treated malaria cases (p = 0.0084) (Figure 5A, Table 2). The number of capillaries per terminal villous (villous vascularity) in the malaria-infected patients was also significantly decreased compared to controls or treated malaria cases (p = 0.0139) (Figure 5B, Table 2).

**Table 2:** Comparison of histoscore variables and measures of villous architecture between different clinical groups

**Variable**	**Controls vs Treated malaria vs Active malaria**	***P. falciparum *** **vs *P. vivax *** **or *Mixed Pf/Pv***	**Subgroups of PCA patients – Treated *Pf *** **Early/Mid or late in gestation**
HISTOSCORE			
Presence of parasitized erythrocytes	<0.001**	1.000	N/A (all –ve)
Inflammation	<0.001**	0.177	0.382
Pigment	<0.001**	1.000	0.471
Parasitaemia	0.0001**	0.855	N/A (all –ve)
Inflammation score (I-III)	0.001**	0.346	0.481
Pigment score (I-III)	0.0002**	0.613	0.335
IMAGE ANALYSIS			
Villous area (mm^2^)	0.0084**	0.321	0.554
Villous vascularity	0.0139*	0.094	0.667
Villi/hpf	0.0001**	0.321	0.235
Perimeter/villous (mm)	0.0006**	0.629	0.203
Total perimeter mm/10 hpf	0.0004**	0.119	0.523

Pf = *P. falciparum*, Pv = *P. vivax*, *P < 0.05, **P < 0.01 N/A = Not Applicable.

The number of villi seen per hpf was significantly increased in both in active and treated malaria cases compared to non-malaria controls (p <0.0001) (Figure 5C, Table 2). The perimeter measurement of individual villi was significantly decreased in malaria cases compared to treated malaria cases or controls (p < 0.0006, Figure 5D, Table 2) but conversely the total perimeter measurement of all villi/10 hpf was significantly higher in both treated and active malaria groups than uninfected controls (p <0.0004, Figure 5E, Table 2).

Villous size and vascularity was reduced in acute placental malaria cases. The number of villi observed per hpf increased significantly in malaria cases even after treatment, and this effect lasted until delivery. However other changes such as the acute decrease in villous size and changes in vascularity, and the histological features of placental malaria, resolved given early detection and appropriate treatment. In addition, although these smaller villi in malaria cases showed a lower perimeter measurement, the total perimeter available for gas exchange per unit area was increased, possibly a compensatory mechanism.

These results are different to those reported by Crocker and colleagues [23], who showed similar villous numbers between non-infected placenta, malaria infected, past infected and active infected placenta, using the histopathological classification system of Bulmer *et al*. [24]. In the patient groups examined in this study, significant changes in villous architecture were seen associated with active malaria infection, which resolved in treated malaria infection, apart from the observed number of villi/hpf.

### Histological scoring of placental pathological changes

The pathological changes associated with placental malaria were scored according to the system of Muehlenbachs *et al*. [22]. The presence of parasites (p <0.001), degree of inflammation (p <0.001) and presence of parasite-derived haemozoin pigment (p <0.001) were all significantly different between active and treated malaria-infected cases. The inflammation score (p = 0.001) and degree of parasitaemia in the placenta (p = 0.0002) were also significantly associated with placental malaria infection (Table 2).

### Analysis of clinical outcomes and villous architecture

There were no significant differences between the demographic details of the different clinical groups (Table 1) other than the maternal haematocrit, which was significantly higher in control cases than malaria treated or infected cases (p < 0.0034). A number of other factors have been previously shown to influence villous size and architecture in other diseases, such as the gestational age of the placenta and placental weight at delivery. There was a small and (after correction for multiple comparisons) probably non-significant decrease in villous size associated with gestational age at delivery (p <0.043).

**Figure 6:**
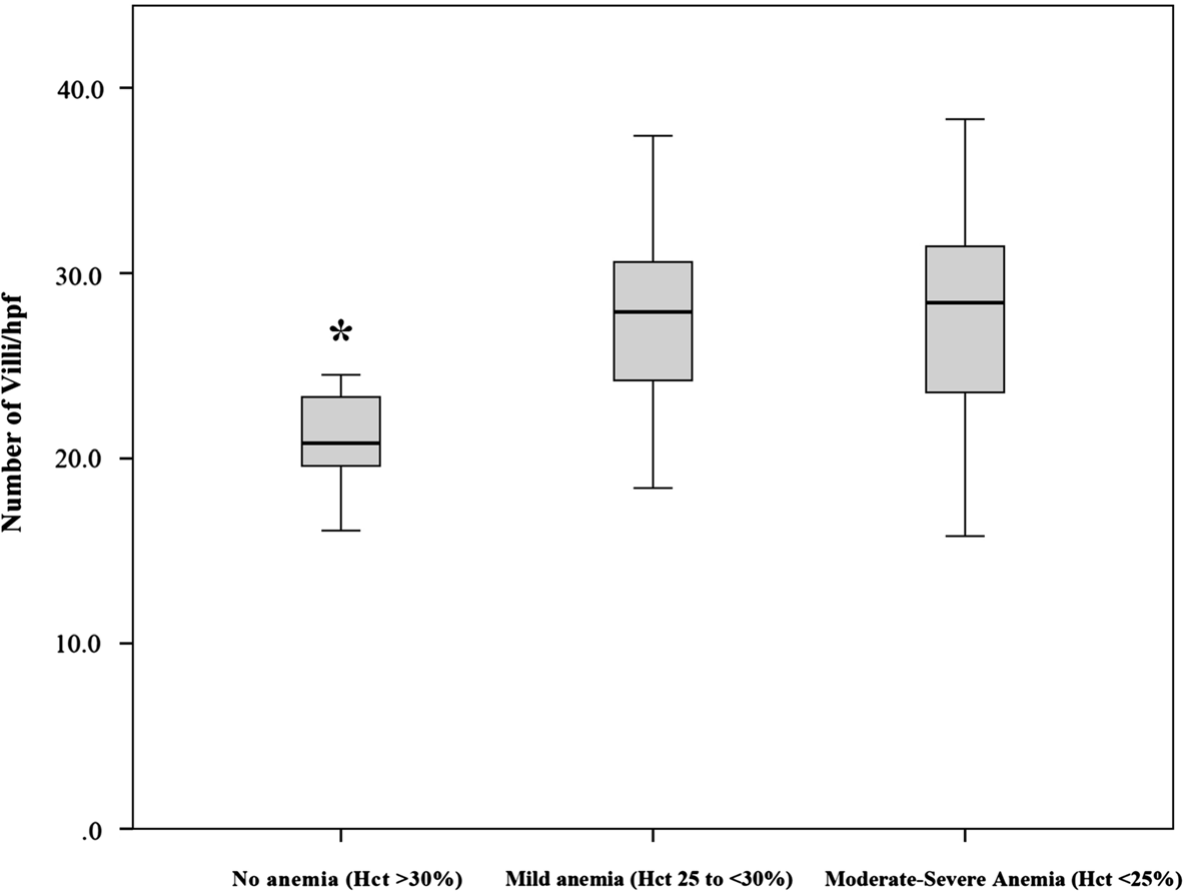
**Boxplot graph of villous number per high-powered field compared to maternal anaemia at the time of infection.** Boxplot showing the significantly higher number of villi seen per 10 hpf in cases with mild and severe anaemia compared to patients without anaemia (*p <0.0034, Fisher’s exact test).

Malaria is the single greatest risk factor for anaemia in pregnant Karen patients and reversal is slow even when malaria and anaemia are treated [19,25]. Because anaemia might cause relative hypoxia within the placenta over a longer period, influencing villous size, the degree of anaemia in the mother (as measured by the haematocrit of the patients at the time of infection) was also correlated with the various measures of villous development above (area, number, vascularity and perimeter measurements). These results are shown in Table 3. There was a significant relationship between the degree of maternal anaemia at the time of infection (whether measured by absolute haematocrit or graded) and the subsequent number of observed villi at delivery, such that patients with moderate to severe anaemia at the time of infection had significantly more villi/hpf at delivery (p = 0.0034) compared to uninfected controls (Figure 6). However no relationship was seen between villous size, vascularity and perimeter and the placental weight, maternal anaemia, gestational age or birth weight. The increase in villous numbers was also seen in treated malaria cases. These results suggest that changes to villous architecture may be dependent not only on malaria infection itself but on concomitant malaria-induced anaemia, and persist despite effective treatment of the acute infection.

**Table 3:** Correlation of villous architecture with clinical outcomes

**Clinical parameter**	**Villous size/μm**^**2**^	**Vascularity (number of vessels/villus)**	**Number of villi/10 hpf**	**Villous perimeter (μm/villos)**	**Villous perimeter (total per 10 hpf)**
Placental weight	0.995	0.547	0.975	0.761	0.208
Gestational Age at delivery	0.043*	0.364	0.247	0.968	0.310
Maternal anaemia Grade^a^	0.656	0.354	0.0034**	0.525	0.214
Maternal haematocrit %^b^	0.142	0.922	0.009**	0.760	0.346
Birthweight	0.833	0.567	0.441	0.454	0.179

^a^Maternal anaemia graded by haematocrit at infection (0 = normal, 1 = 25-30%, 2 = <25%, see Methods).

^b^% maternal haematocrit at infection.

Figures in bold are significant at *P<0.05 and **P<0.01.

### Histology and placental villous architecture are similar in *Plasmodium falciparum* or *Plasmodium vivax*

The relationship between villous architectural changes and the type of malaria infection was examined in malaria cases, for both monospecies infections with *P. falciparum* (n = 26) or *P. vivax* (n = 5), and where patients had both species either on separate occasions during gestation or mixed infection with both (n = 9). No significant differences were seen between any of the placental architectural parameters and either mono-infection with either *P. falciparum* or *P. vivax,* or in pregnancies where both species had been found, either as a mixed infection or on separate occasions (Table 2). The comparison of the morphological changes in the placenta in this study is however necessarily limited by the small number of samples from *P. vivax* patients, which could generate a bias in the analysis.

Histological descriptions of pure *P. vivax* placental malaria are so far limited [17,26-28]. Patients who had sequential (not simultaneous) *P. falciparum* and *P. vivax* infections occurring at different times in the same gestation appeared similar histologically, whichever infection was first, without differences in the degree of inflammatory response. Whether multiple recrudescent infections have clinically or pathologically different results from mono- or true dual infection remains to be determined.

### Relationship between timing of infection and delivery

In PCA patients, all of whom had mono-infection with *P. falciparum*, selection was according to the timing of the last treated malaria infection compared to delivery. These groups were divided into ‘early’ (n = 6 women who had a clinical episode of malaria treated at least five months prior to delivery and no subsequent infections); ‘middle’ (n = 6 included those with malaria infection two to four months before delivery) and ‘late’ infection (n = 5 those with treated malaria infection within one month before delivery). Comparison showed no significant differences between timing of infection before delivery and any of the parameters of the histoscore or villous architecture (Table 2), although the groups were small.

Because pathology at a given point in gestation can cause placental insufficiency, fibrinoid necrosis and scarring, subsequent development may be affected even if the infection is treated, and still be seen histologically at delivery some time later. However when groups of patients within the PCA study were compared, all of whom had been infected, diagnosed and treated for malaria but at varying times in gestation before delivery, there were no differences in villous architecture or resultant histoscore in the placenta at delivery. The similarity of both villous architecture and histoscore of treated placental malaria with normal control levels implies that if malaria infection is detected and treated effectively, the placenta retains enough compensatory mechanisms to return to normal growth and development without long-term pathological changes caused by untreated malaria infection.

The acute reduction of villous size and perimeter associated with active malaria infection seems unlikely to reflect decreased blood flow in the placenta before delivery (for instance due to the effects of vascular obstruction by sequestered parasites, leukocytes or fibrin deposition) because these processes affect the maternal blood space. Changes in villous vascularity and size in other diseases examined using morphometry developed over a longer period due to poor oxygenation and hypertension, leading to defective stem villous development and hence vascularization of terminal villi [16,29,30]. Apart from blood flow, the activity of various growth-promoting hormones, such as insulin-like growth factor or placental growth factor, plays a role in supporting normal placental development [31], but changes in these growth axes would not explain the acute shrinkage seen in malaria infection. This study specifically examined changes in terminal villi only, and therefore excluded areas that may be affected by other pathological changes caused by placental malaria infection, such as infarction or extensive intervillous fibrin deposition. Such changes also have an important effect on placental function and the extent of these lesions is another important factor affecting the structure and function of the remaining placenta.

Villous size was interdependent with villous vascularity. If there was a generalized decrease in villous vessel numbers in malaria infection this may cause an acute shrinkage of villous area, which seems more likely than a sudden loss of established villous blood vessels. Placental malaria infection causes acute changes to both maternal and foetal haemodynamics, which reverses rapidly, within three days, after effective treatment [32,33]. Foetal heart rate is increased and it may be that other physiological parameters of the foetal-maternal circulation in the placenta, such as blood pressure, are altered in acute infection. Whether this reduces the perfusion pressure of the villi or causes changes to villous size, which are maintained and seen on subsequent histology, is unclear. Ultrasound Doppler investigation of the placenta during acute malaria also shows that malaria is associated with significantly smaller placental volumes, which can be linked to subsequent IUGR and poor outcome [34].

The pathophysiological effects of malaria infection in the placenta include inflammatory cell localization, cytokine release and direct effects on villous function by parasitized erythrocyte binding [35-38]. Cytokine and chemokine release, induced in host leukocytes and syncytiotrophoblastic cells and enhanced by phagocytosis of malaria pigment [39], causes auto-induction of chemokines and angiogenic factors which can attract host monocytes [40]. In other organs where PRBC and leukocytes are sequestered, signalling across endothelial monolayers causes changes to interendothelial cell junctions and oedema formation, such as in the brain during cerebral malaria [41]. There is evidence that adhesion of PRBC to syncytiotrophoblastic cells initiates signalling in these cells by tyrosine phosphorylation [42]. The current findings suggest another potential mechanism for placental dysfunction during malaria infection, where the presence of PRBC and host leukocytes reduces villous surface area, essential for foeto-maternal transfer of gas or nutrients, and disposal of potentially toxic metabolites, affecting foetal growth and development in undiagnosed or recurrent infections. This may result from either increased external osmotic pressure on the villus, or changes to foetal and maternal blood pressure reducing vascular filling, causing acute shrinkage. However the increase in relative villous numbers and therefore total available villous perimeter per unit area, which persists after treatment of infection through to delivery, hints at an attempt at compensation for this.

## Conclusions

Digital image analysis was used to examine pathological changes to villous architecture in patients with active and treated malaria and controls. Villous area was significantly correlated with vascularity and inversely correlated with density of villi per unit area. UK controls showed significantly greater villous area and vascularity than Karen groups. There was a significant relationship between villous number and anaemia at the time of infection, but not placental weight and gestational age at delivery. Active malaria-infected cases showed significantly smaller villous area, vascularity, density, and perimeter measurement than controls. However increased villous numbers in malaria cases caused a compensatory increase in total available villous perimeter. Histology score and villous architecture showed no significant differences between infections with either *P. falciparum*, *P. vivax*, or consecutive infections by both during the course of pregnancy. These results imply that villous architecture changes acutely during malaria infection, causing shrinkage of the terminal villous and compensatory increase in the number of villi which persists following treatment and eradication of the infection. It cannot be excluded that the reversal of placental morphological changes may be related to improvement in maternal anaemia, caused by malarial treatment. However other architectural and pathological changes induced by malaria infection are reversible given early detection and appropriate anti-malarial treatment.

## Competing interests

The authors declare that they have no competing interests.

## Authors' contributions

SC, AM, EP, and GDT performed the experiments, analysed data and wrote the paper. UC, EP, PV, RM, MJR, and FN collected samples, helped analyse data and wrote the paper. SC and SJL performed statistical analysis. GDT, UC, EP, RM, MJR, and FN conceived of the study and designed experiments. All authors read and approved the final manuscript.
